# Rapid Detection of *Epinephelus* Species Substitution in the Greek Market Using High-Resolution Melting Analysis

**DOI:** 10.3390/genes16030255

**Published:** 2025-02-22

**Authors:** Evanthia Chatzoglou, Nefeli Tsaousi, Ariadni Spetsieri, Emmanouil E. Malandrakis, Helen Miliou

**Affiliations:** Laboratory of Applied Hydrobiology, Department of Animal Science, School of Animal Biosciences, Agricultural University of Athens, 11855 Athens, Greece; tsaousinefeli@gmail.com (N.T.); stud316088@aua.gr (A.S.); emalandrak@aua.gr (E.E.M.); elenmi@aua.gr (H.M.)

**Keywords:** mtDNA, barcoding, *Epinephelus*, HRM, fish mislabeling, fraud, *COI*, *16SrRNA*, *cytb*, *ND2*

## Abstract

**Background/Objectives**: Fish are vital in the Mediterranean diet, offering protein, nutrients, and ω-3 fatty acids. Greek consumers favor wild-caught, high-value fish like the dusky grouper (*Epinephelus marginatus*) classified as “vulnerable” and the white grouper (*Epinephelus aeneus*) classified as “near threatened” species, according to the IUCN Red List. Due to their premium prices and complex supply chains, these species are susceptible to fraud, especially through mislabeling. This practice not only deceives consumers but also poses health risks and encourages illegal fishing. DNA-based methods have shown effectiveness in accurately identifying species, even in processed samples. The aim of this study is to apply high-resolution melting analysis (HRM) as a rapid, effective method for monitoring the appropriate labeling of the two *Epinephelus* species in the Greek market. **Methods**: In this study, fresh fish from Greek catches as well as cooked, frozen, and filleted samples collected from the Greek market were identified using DNA barcoding. HRM analysis based on single nucleotide polymorphisms (SNPs) was used to differentiate between locally sourced *E. marginatus* and *E. aeneus* from their imported counterparts or from other species available in the Greek market that could be used in substitution incidents. **Results:** Using HRM analysis, cases of species mislabeling were identified and were also confirmed using sequencing. **Conclusions:** HRM analysis proved to be an accurate and cost-effective method for rapidly processing a large number of samples; therefore, it could serve as a valuable tool in extensive market controls as well as for bio-diversity conservation monitoring.

## 1. Introduction

Fish play an important role in the Mediterranean diet, providing high-quality protein content, vitamins, minerals, and large amounts of ω-3 polyunsaturated fatty acids. Although aquaculture products cover a large percentage of fish production, consumers still have a preference for wild catches. *Epinephelus* is a genus of predatory fish, including 89 recognized species, found in seas around the world. They are among the highest-priced species in fish markets, and only a few species are used in aquaculture [1]. Two of the most common representatives in Greece are the dusky grouper *E. marginatus*, a reef-associated species distributed in the Mediterranean Sea and Atlantic and Indian Ocean, and the white grouper *E. aeneus*, a demersal species distributed in the Mediterranean Sea and the Eastern Atlantic Ocean [2]. According to the IUCN Red List of Threatened Species, the *E. aeneus* it is classified as a “near threatened” species [3]. It is a promising candidate fish for intensive aquaculture due to its high commercial value, excellent taste, fast growth rate, increased hardiness, and disease resistance. The first breeding attempt of *E. aeneus* in captivity was in the late 1990s; however, to date, not much progress has been made [3,4].

*E. marginatus* is a widely distributed species that inhabits rocky reef habitat. Its global population is comprised of at least four mostly separated subpopulations. It has been heavily targeted by recreational and commercial fishers, and it also supports dive ecotourism especially in the Mediterranean Sea and southeastern Africa. According to the IUCN Red List of Threatened Species, *E. marginatus* has been classified as “vulnerable” worldwide [5]; however, especially in the European and Mediterranean regions, its population is known to be greatly reduced in certain areas and has been classified as “endangered” [6]. The slow growth rate and unique mode of reproduction of *E. marginatus* have significant effects on its population dynamics and vulnerability.

Both species are distributed in Greek markets and restaurants, with high demand, especially during the summer period. According to a European Union survey, Greek consumers prefer fresh fish from domestic fisheries, compared to farmed, frozen or canned fish, while in many cases the price/quality ratio is the main factor influencing consumers’ choice [7]. European food regulations enforce strict traceability standards, requiring product labeling across all EU countries as mandated by Regulation (EU) No. [8]. These labels must clearly state details such as the product’s origin, species, catch or farming method, and production process. The seasonal nature of Greek small-scale fisheries (SSFs), along with declined fish stocks and imported *E. marginatus* and *E. aeneus* in the Greek market, raises the question of authenticity, traceability, and proper labeling of *Epinephelus* fish from Greek catches.

These large fish are sold whole or filleted (fresh or frozen) and processed (dried, salted, smoked, canned, etc.) in markets as well as cooked in restaurants. Under these conditions their morphological characteristics change, making species identification difficult, increasing the risk for fraudulent acts [9,10]. Incidents of grouper fillet mislabeling or substitution by cheaper fish species have been reported in the literature. For example, *E. aeneus* has been substituted by the Nile perch (*Lates niloticus*) or the wreckfish (*Polyprion americanus*) [11]; *E. marginatus* by *Epinephelus itajara* [12], *Argyrosomus japonicus*, *Pollachius virens*, and Nile perch (*L. niloticus*) in Spain [13], as well as other groupers by *Pangasianodon hypophthalmus* [14]. Molecular techniques based on DNA, e.g., *COI* barcoding, are proven to be effective for accurate and robust fish species identification [15], using samples of any state: raw, cooked, processed, frozen, etc. Several studies were reported for the *Epinephelus* genus [16], and some of them emphasized the potential health risks of consuming mislabeled fish [17], especially those that might contain harmful contaminants or toxins [18]. One particular study found that 31% of 975 fish samples were mislabeled, with some of the substituted species being prone to ciguatera poisoning. The study also highlighted the effectiveness of DNA barcoding in species identification and assessing health risks. For example, among the mislabeled *Epinephelus *malabaricus** samples, two yellow-edged lyretails (*Variola louti*) and three leopard coral groupers (*Plectropomus leopardus*), were found, and both of these species are at risk of ciguatera poisoning [19]. This emphasizes the potential health risks of consuming mislabeled fish, especially those that might contain harmful contaminants or toxins.

Although sequencing-based methodologies are accurate, they can be time-consuming and not applicable for rapid inspections in the market [9]. For quick, on-site species identification using fish tissue, a RT-NASBA (real time nucleic acid sequence-based amplification assay) device could be used [20].

Other non-sequencing-based techniques such as PCR-RFLP are widely used in the identification of fish species [21,22,23]; however, they require the presence of informative restriction sites for species discrimination and a well amplified PCR product. In addition, these methods can be time consuming as they require several steps for the analysis, limiting the number of samples analyzed per day [24]. Alternative, non-sequencing-based techniques such as real time PCR and HRM analysis appear to be more efficient in fish species identification for phylogenetic studies or market control [24,25,26,27,28].

In this study, mitochondrial DNA amplicons of *E. aeneus* and *E. marginatus* were used to distinguish *E. aeneus* and *E. marginatus* captured in the Greek seas from other fish species that could be used in fraudulent activities as well as from their imported counterparts found in the Greek market. We employed non-sequencing techniques, such as high-resolution melting (HRM) analysis, along with morphological identification (when applicable) and DNA barcoding to confirm species identification and to parameterize and validate the methods used. HRM is a method with high sensitivity due to the use of high concentration saturation dyes, making it possible to discriminate between amplicons of a single base [29,30], thus making it possible to discriminate even closely related species. The single-step procedure is performed using one close-tube analysis method, and the complete overlap of sample curves from identical sequences offers the possibility for rapid analysis of a large number of samples per day [24,29]. Given these advantages, the use of HRM in fish species identification presents a rapid and reliable approach for distinguishing *E. aeneus* and *E. marginatus* in Greek catches as it could be applied in large-scale market controls, effectively preventing fraud and ensuring the authenticity of seafood products.

## 2. Materials and Methods

### 2.1. Sample Collection and Morphological Identification

**Figure 1:**
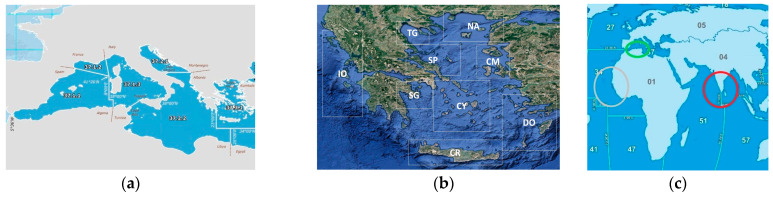
(**a**) Division captions according to FAO major fishing areas [31]; 37.2.2 Ionian; 37.3.1 Aegean; 37.1.1 Balearics. Credits: FAO, 2024; (**b**) Sampling areas of Serranidae specimens from Greek seas; CM: Chios-Mytilene, CR: Crete, CY: Cyclades, DO: Dodecanese, IO: Ionian Sea, NA: North Aegean Sea, SG: Saronic Gulf, SP: Sporades, TG: Thermaikos Gulf; Credits: Google Map; (**c**) Samples with indicated origins from Senegal (grey circle), Tunisia (green circle), and India (red circle). Map: FAO, 2024 [31].

The specimens examined were samples of (a) *E. aeneus* and *E. marginatus* of Greek fish catches in FAO subareas 37.2 (Ionian Sea division 37.2.2) and 37.3 (Aegean and Cretan Seas), as shown in Figure 1a,b; (b) samples from the Division captions in 37.1.1 Balearics (Figure 1a); (c) imported fish labelled as *E. aeneus* and *E. marginatus* purchased from fish markets and supermarkets with the fishing area indicated (Figure 1c); (d) fresh and cooked fish collected from restaurants served as *E. aeneus* or *E. marginatus*; and (e) other species closely related to *Epinephelus* sp., such as *Epinephelus costae* either from fish catches or imported; and (f) species that have either been cited in the literature to be used in *Epinephelus* sp. substitution events or are sold filleted at low price (as *Thunnus thynnus*) and are available in the Greek market.

When morphological identification of samples was feasible, such as in the case of fish from Greek catches or fresh imported samples stored on ice, species identification was conducted based on their morphological characteristics (e.g., body shape, fins, rays, and coloration) according to FAO [32]. Samples were categorized into groups according to their fishing area (for Greek catches) or the origin indicated by the seller (for market-purchased samples). For frozen, filleted, and cooked samples, morphological identification was not possible. The list of all samples used in this study is shown in Table 1, along with details for their collection sources. For the purposes of this study, selected samples from all species were cooked (roasted or boiled in soup with other ingredients) or frozen and were used for comparisons between different states for validation of the HRM method.

After collection and morphological identification when applicable, various tissues (muscle, liver, fins, and gills) were collected from all samples. These tissues were either used directly for DNA extraction or preserved in 70% ethanol and stored at −20 °C for use in subsequent downstream applications.

### 2.2. DNA Extraction, PCR, and Sequencing

For each sample, 25 mg of tissue (fresh, frozen or cooked) was dissected using sterile, disposable plastic forceps, suspended in PBS, and homogenized using a cordless motorized pellet pestle (Kimble, Millville, NJ, USA). Total DNA extraction was performed using either the NucleoSpin^®^ Tissue (Macherey-Nagel, Düren, Germany) kit for all fresh tissues and/or the NucleoSpin ^®^ Food (Macherey-Nagel, Düren, Gernamy) kit according to the manufacturer’s instructions for all types of samples (fresh, frozen, and cooked). Total DNA concentration and quality were measured using the Biophotometer D30 (Eppendorf, Hamburg, Germany) and electrophoresed on an 0.8% agarose gel. The quality of DNA was assessed using both DNA extraction protocols, and the results were compared. High-quality DNA (260/280 ratio of 1.8–2.0 and 260/230 ratio of 2.0–2.2) from either extraction protocol was selected for downstream PCR applications.

Primers used for the amplification of mtDNA regions were either sourced from the literature or designed *de novo* in this study using the specialized software Geneious Prime 2023.0.3 [33] based on the complete mtDNA of *E. aeneus* (accession number LC545417.1) [34], *Epinephelus moara* (accession number NC_017891.1), *Mycteroperca bonaci* (accession number OP035077.2), *Epinephelus stictus* (accession number NC_021133.1), and *Epinephelus bruneus* (accession number FJ594964.1) as well as partial sequences of *E. marginatus* (accession numbers AM158300.1, NC_087998.1, and KC500686.1). For *COI* barcoding, the primers used, namely, m*COI*Fu and m*COI*Ru, were modified by the validated study for the generation of barcodes [35] and were tailed with the universal primers M13 uni −21) and M13 rev (−29), respectively. For the amplification of *16S rRNA* and *12S rRNA*, new primers for *ND5* and *ND2* mtDNA regions were designed with Geneious Prime^®^ 2023.0.3. PCR reactions were performed using the KAPA Taq PCR Kit (KAPA BIOSYSTEMS, Cape Town, South Africa) in a MiniAmp Plus Thermal Cycler (Applied Biosystems Foster City, CA, USA). In total, 200 ng of total DNA were used as a template. PCR cycles were designed as follows: initial denaturation at 95 °C for 3 min and amplification for 40 cycles with an elongation duration of 1–2 min depending on the fragment’s length and annealing temperatures as shown in Table 2. After the completion of the amplification cycles, a final elongation step at 72 °C for 10 min was applied.

PCR products were run on 1–2% agarose gels and stained with Midori Green (Nippon Genetics, Tokyo, Japan).

### 2.3. Sanger Sequencing and In Silico Analysis

All PCR products were sequenced for both strands using the Sanger dideoxy method (EUROFINS Genomics sequencing services) with the universal primers M13 uni (-21) and M13 rev (-29) for the COI barcode or the gene specific primers shown in Table 2.

The sequences obtained were identified using the COI barcode sequence with Blast search tool [38] showing >99% identity for 100% query cover and an E-value < 0.01 when compared with the mitochondrial complete genome reference sequences or partial isolates of each species from GenBank [39] and the BOLD database [40]. To identify SNPs and assess the degree of homology within or between species, all samples from this study, along with sequences retrieved from the databases, were aligned using Clustal Omega [41], via EMBL-EBI Job Dispatcher Sequence Analysis Tool [42]. Based on the comparative analysis of the studied gene regions, species-specific polymorphic sites were identified. New primers were designed with Geneious Prime 2023.0.3 [33] to amplify smaller fragments of 100–150 bp, which were subsequently used in high-resolution melting (HRM) experiments.

### 2.4. HRM Analysis

To define a suitable region to be used as a species-specific marker for HRM analysis, the sequenced regions of the *COI* barcode, *cytb*, *16S r*RNA, and *ND2* were selected, as they are often used in phylogenetic studies [10,43,44,45]. Sequences of the samples in current study and databases (*E. aeneus*, *Epinephelus undulosus* (NC_071920.1), *Epinephelus poecilonotus*, *E. marginatus*, *L. niloticus*, *Argyrosomus regius*, *P. hypophthalmus*, *P. americanus*, and *T. thynnus*) were aligned to locate SNPs that could discriminate between species and/or populations within species. Based on these comparisons, regions that could serve as templates for the amplification of smaller fragments (113–156 bp) were defined. For the amplification of the selected regions, specific primers were designed (Table 3) that are able to anneal with all species of the Epinephelidae family as well as with species frequently used in substitution events, such as the Nile perch (*L. niloticus*), the wreck fish (*P. americanus*) [11,13], and *P. hypophthalmus* [14]. PCR conditions were optimized to ensure successful amplification across all samples. Various DNA template concentrations (ranging from 1 to 10 ng) were tested, with the optimal concentration determined to be 8 ng. Primer efficacy and specificity were verified with conventional PCR using the annealing temperatures listed in Table 3, followed by analysis with gel electrophoresis on a 2% agarose gel.

HRM reactions were performed with the KAPA HRM FAST qPCR Kit (KAPABIOSYSTEMS Cape Town, South Africa) [46]. Each 20 μL reaction contained the KAPA HRM FAST Master Mix with EvaGreen^®^ dye. Reactions were performed, and the reaction contained 8 ng of total DNA, 2.5 mM MgCl_2_, and 300 nM of each primer. HRM reactions were carried out using a CFX96 Real-Time PCR thermocycler (Bio-Rad Laboratories, Inc. Hercules, CA, USA) as follows: enzyme activation at 95 °C for 2 min, 45 cycles of amplification with denaturation step at 95 °C for 5 s, annealing step with temperatures according to the set of primers used as shown in Table 4 for 20 s, and elongation step at 72 °C for 10 s. Melting curve dissociation was performed using 0.2 °C increments from 65 °C to 95 °C as previously described [24]. Curve data analysis was performed with Bio-Rad Precision Melt Analysis Software, version 1.2 (Bio-Rad Laboratories Inc. Hercules, CA, USA) [47]. To enhance the accuracy of identifying variations in DNA sequences, two cluster classification parameters, including the Melt Curve Shape sensitivity (which defines the stringency level for classifying melt curves into distinct clusters) and Tm difference threshold (which defines the minimum difference in the melting temperatures (Tm) required to distinguish between two DNA samples), were adjusted for each gene to obtain maximum confidence percent values. The curve analysis parameters for each tested fragment were optimized using samples of known sequences as a reference for each studies species (*E. aeneus* or *E. marginatus*). Final parameter values were determined when the confidence percentage for species clustering reached 95–99.9%. All samples were sequenced either prior to or following HRM analysis to verify the results.

## 3. Results

### 3.1. Species Identification by Morphological Characteristics

**Figure 2:**
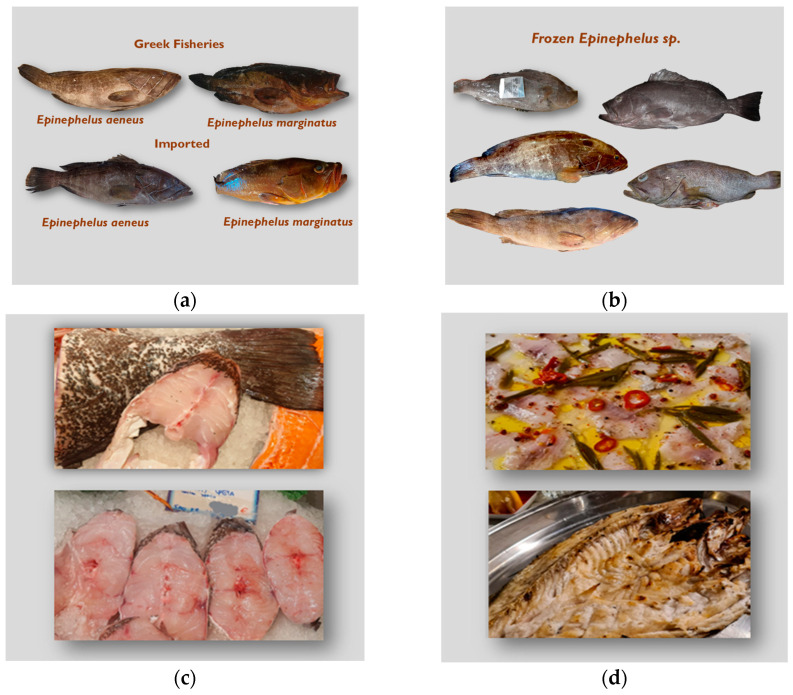
Representative samples of *Epinephelus* collected for this study. (**a**) Examples of fresh *E. aeneus* and *E. marginatus* samples from Greek catches and from the Greek market; (**b**) frozen samples sold as *E. aeneus* and *E. marginatus* in the Greek market; (**c**) filleted samples sold as *E. aeneus* and *E. marginatus* in the Greek market; (**d**) cooked samples served at Greek restaurants.

Fish from Greek catches (Figure 2a) were identified based on their morphological characteristics, including body shape, fins, rays, and coloration, following FAO guidelines [32]. The samples were further grouped according to their geographic origin (Table 1). *E. aeneus* was identified morphologically by the presence of three spines and two or three pale lines on the gill cover [32,48]. Similarly, the morphological identification of *E. marginatus* was based on the presence of three spines on the gill cover, along with a rounded caudal fin featuring a distinctive white line at its tip [48].

Morphological identification of imported fish samples purchased from the fish market was feasible only when the fish were fresh (i.e., stored on ice; Figure 2a). On the contrary, frozen samples sold as *E. aeneus* were unidentifiable due to significant alterations in key morphological characteristics during freezing (Figure 2b). During freezing, ice crystals formed within the tissues and altered the fish’s external features, while frost buildup further obscured key characteristics. Additionally, the freezing process made the fins more fragile, causing them to break, become misshapen, or even merge with the body due to tissue rigidity, making it difficult to count them accurately. Similarly, morphological identification was not possible for filleted samples (Figure 2c) or the majority of cooked samples obtained from restaurants (Figure 2d) that were labeled as either *E. aeneus* or *E. marginatus*. These samples were therefore classified as “unknown” for subsequent analyses.

### 3.2. Sequencing-Based Identification—COI Barcoding

DNA barcoding identified all Greek-caught fish samples and eight imported samples (from Senegal, Tunisia, and Spain) as *E. aeneus*. A primary barcode exhibited 100% identity with *E. aeneus* sequences from Turkey (KT805239.1), the eastern Atlantic Ocean (LC545417.1) [34], MN729687.1 [49], and Senegal KM077913 [18]. A secondary barcode, differing by a single nucleotide polymorphism (SNP), was found in one sample from the central Aegean Sea. Two barcodes were found among imported *E. aeneus* samples purchased at Greek fish markets: one identical to the Greek-caught samples and another with a single SNP. Two frozen, imported samples from India, sold as “white grouper”, were identified as *E. undulosus* and *E. poecilonotus*. Finally, two restaurant samples also served as “white grouper” were identified as *A. regius*.

All morphologically identified *E. marginatus* samples, both from Greek catches and imports (Senegal, Tunisia, and Spain), were confirmed by DNA barcoding. A single, primary barcode was identified for *E. marginatus* samples from Greek fisheries, matching existing genetic codes for *E. marginatus* from the Mediterranean [50,51] and the Atlantic [52]. Additionally, three barcodes presenting different SNPs were determined for unique samples from the Saronic Gulf, Central Aegean and Ionian Seas, showing 100% identity with samples from Tunisia and Brazil [53]. Additionally, all samples from other species collected in the Greek market (Table 1) were identified by barcoding to be used in downstream HRM applications.

The obtained sequences showing different SNPs for each sample obtained from different fishing areas or in the fish market and the regions studied (*COI* barcode, *16S rRNA*, *cytb*, CR, and *ND2*) were submitted to GenBank. Their accession numbers, along with their percent identities (for 100% query cover and an E-value < 0.01) when compared to mitochondrial complete genome reference sequences or partial isolates of each species from GenBank, are presented in Table 4.

### 3.3. Sequencing of mtDNA Candidate Regions for the Detection of SNPs

To identify a suitable target region for high-resolution melting (HRM) analysis, regions of the mitochondrial genome were sequenced. This sequencing aimed to identify single nucleotide polymorphisms (SNPs) capable of differentiating samples both between and within species. The selected regions were the partial sequences of *16S* rRNA, *12S* rRNA, *cytb*, *ND5*, *ND2*, and CR. Sequences were aligned, and SNPs were detected between species and within species.

For *E. aeneus*, all samples had identical sequences for *16S* rRNA, *12S* rRNA, *ND2,* and *ND5* regions, showing 100% identity to the reference sequence LC545417.1. In contrast, the *cytb* region revealed three single nucleotide polymorphisms (SNPs) across different samples, defining three haplotypes. The first haplotype (Ea1) was predominant in Greek catches but was also found in samples from Tunisia and Spain (both from this study and GenBank) and one sample from Senegal (this study). The second haplotype (Ea2) matched LC545417.1 and was present in samples from Senegal and Tunisia. The third haplotype (Ea3) was found in four samples from Greek catches. Consequently, no clear correlation was observed between these *cytb* haplotypes and the geographic origin of the samples. Analysis of the control region (CR) revealed high heterogeneity, with SNP insertions and deletions indicating distinct haplotypes within the Greek samples.

For *E. marginatus*, the *16S rRNA* sequences from the majority of samples from Greek catches exhibited 100% identity with samples from Senegal (this study) and Brazil (NC_087998.1). A new haplotype was identified in a single sample from Greek catches, while another new haplotype was observed in one sample from Senegal. Regarding the *cytb* gene, most samples caught in Greece shared a predominant haplotype (Em1), which showed 100% identity with samples collected in Greece (EU264008.1) and the western Mediterranean. One sample had a unique SNP (Em2), while three samples shared a haplotype (Em3) identical to samples from Tunisia, Spain, the Canary Islands, South Africa, and Brazil. The *ND2* region showed one main haplotype in Greek-caught samples and three additional haplotypes, each differing by a single SNP in separate samples. Notably, imported *E. marginatus* samples from the Greek market exhibited four distinct SNPs, none of which matched the haplotypes found in Greek catches. The control region (CR) showed high similarity within Greek-caught samples, with two haplotypes differing by a single SNP. However, several differences were observed when compared to the Brazilian sample (NC_087998.1).

### 3.4. HRM Analysis of Mitochondrial Gene Regions for the Identification of E. aeneus and E. marginatus Species

Analysis of sequencing data revealed conserved nucleotide regions, which were then used to design new primers (Table 4). To provide comparative data, samples of Nile perch (*L. niloticus*), meagre (*A. regius*), Atlantic bluefin tuna (*T. thynnus*), striped catfish (*P. hypophthalmus*), and wreckfish (*P. americanus*) were collected from Greek fish markets and supermarkets. Importantly, both raw and cooked samples of these species were analyzed and directly compared to assess the impact of cooking on DNA integrity and subsequent analyses. To detect substitutions of *E. aeneus* and *E. marginatus* from Greek fisheries compared to samples listed in Table 1, high-resolution melting (HRM) analysis was performed on four mitochondrial DNA fragments: two protein-coding genes (*cytb* and *ND2*), a ribosomal RNA gene (*16S rRNA*), and a portion of the highly variable control region (CR). All four regions were analyzed for *E. aeneus*, while only the *ND2*, *16S* rRNA, and CR regions were analyzed for *E. marginatus* (*cytb* was excluded for this species). To achieve maximum confidence levels, the melting curve shape sensitivity and Tm difference threshold for each region studied. These parameters were either selected automatically by the software or were adjusted manually to achieve confidence levels from 95.0 to 99.9% for all samples as shown in Table 5. The details of the samples analyzed (i.e., species, and raw or cooked status) as well as the percent confidence level for each sample is provided in Figures S1 and S2 of the Supplementary Materials. In each of the above analyses, cooked samples consistently clustered with the respective raw (fresh or frozen) fish, demonstrating the robustness of the method even with processed samples.

#### 3.4.1. HRM Analysis for the Detection of *E. aeneus* Species

**Figure 3:**
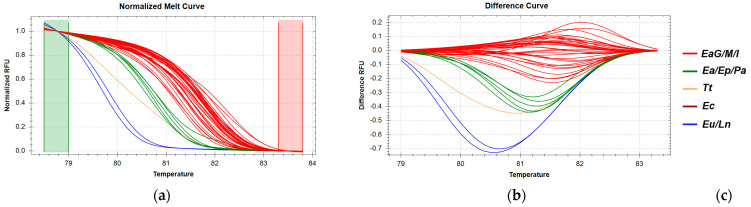
HRM reactions for the discrimination of *E. aeneus* from Greek fisheries compared to other species sold in the Greek market using the *16S rRNA* fragment: (**a**) Normalized melt curves from HRM analysis; (**b**) Difference curves from HRM analysis; (**c**) Species in clusters; Ea: *E. aeneus*, Eu: *E. undulosus*, Ep: *E. poecilonotus*, Ec: *Epinephelus costae*, *Ln: L. niloticus*, *Tt: T. thynnus*, *Pa: P. americanus*; G: fish caught in Greek seas, I: imported in Greek markets, M: fish caught in the Mediterranean Sea.

The *16S rRNA* gene region, showing 100% sequence identity across all *E. aeneus* samples from Greek catches (EaG) and the Mediterranean Sea, also exhibited identical sequences in the imported *E. aeneu*s samples (EaΙ) from the Atlantic Ocean. HRM analysis of this region revealed that the majority of *E. aeneus* samples clustered together with high confidence (Figure S1a), forming a distinct group. However, a small subset of *E. aeneus* samples clustered with *E. poecilonotus* and *P. americanus* (Figure 3), indicating that this particular *16S* rRNA region, while useful for some differentiation, lacks the resolution to fully distinguish all *E. aeneus* samples from other species in this analysis.

**Figure 4:**
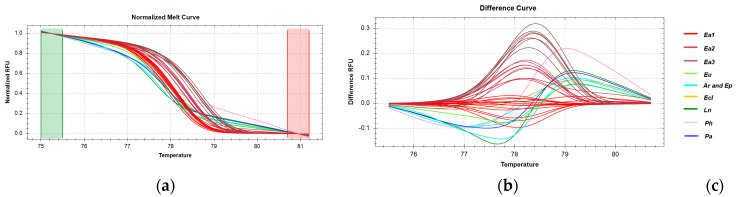
HRM reactions for the discrimination of *E. aeneus* from Greek fisheries compared to other species sold in the Greek market using the *cytb* fragment: (**a**) Normalized melt curves from HRM analysis; (**b**) Difference curves from HRM analysis; (**c**) Species in clusters; Ea: *E. aeneus*, Eu: *E. undulosus*, Ep: *E. poecilonotus*, Ar: *A. regius*; Ln: *L. niloticus*, Ph: *P. hypophthalmus,* Pa: *P. americanus*. G: fish caught in Greek seas, I: imported in Greek markets.

For *cytb*, the melt region was adjusted manually and is shown in Figure 4a. Τhe melting curve shape sensitivity and Tm difference threshold were set manually (Table 5). Under these conditions, confidence levels ranged from 94.4 to 98.5% for the Ea clusters (Figure S1b). The region selected for HRM analysis includes the SNPs found in different haplotypes of *E. aeneus* samples by sequencing. As shown in Figure 4b, Ea samples (including samples from Greek catches, the Mediterranean and the Atlantic Ocean) are grouped in three different clusters, with each one corresponding to a different haplotype. When this fragment is used, all Ea samples are distinctly separated from other species (Figure 4b). The two samples served in a restaurant as *E. aeneus* but identified by barcoding as *A. regius* are clearly grouped in a different cluster with *E. poecilonotus.*

**Figure 5:**
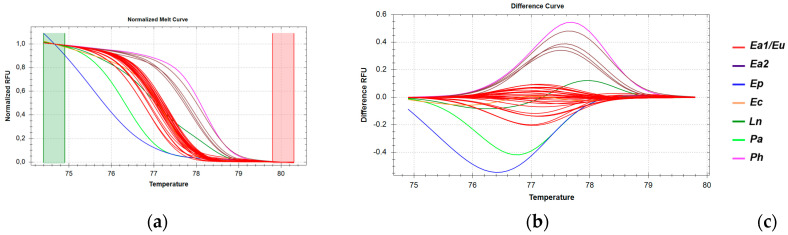
HRM reactions for the discrimination of *E. aeneus* from Greek fisheries compared to other species sold in the Greek market using the CR fragment: (**a**) Normalized melt curves from HRM analysis; (**b**) Difference curves from HRM analysis; (**c**) Species in clusters; Ea: *E. aeneus*, Eu: *E. undulosus*, Ep: *E. poecilonotus*, Ec: *E. costae*; Em: *E. marginatus*; Ar: *A. regius*; Ln: *L. niloticus,* Pa: *P. americanus*, Ph: *P. hypophthalmus.*

For the CR fragment analysis, the melt region was automatically set, and all other parameters were adjusted to achieve maximum percent confidence levels (Figure S1c). *E. aeneus* samples were grouped in two clusters (Figure 5). This result was expected as sequencing has revealed one SNP in the region studied. However, both clusters include samples from Greek catches, the Mediterranean Sea, and the Atlantic Ocean, indicating that there is no direct correlation between a haplotype and the geographic origin of the samples. Additionally, when using this fragment, *E. undulosus* is erroneously clustered within the *E. aeneus* group. This result was constant, even when different PCR conditions were used or different analysis parameters were applied. Alignment of this fragment shows that the *E. undulosus* collected in Greek market shows 92.50% identity with the reference mitochondrial genome of *E. undulosus* including gaps, indicating high variability of this species.

**Figure 6:**
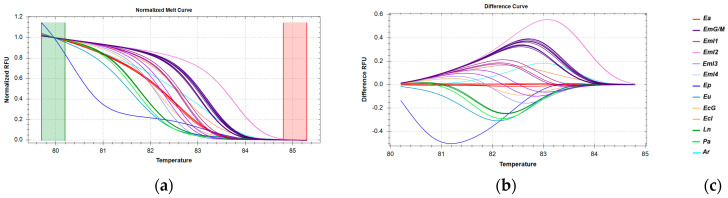
HRM reactions for the discrimination of *E. aeneus* from Greek fisheries compared to other species sold in the Greek market using the *ND2* fragment: (**a**) Normalized melt curves from HRM analysis; (**b**) Difference curves from HRM analysis; (**c**) Species in clusters; Ea: *E. aeneus*, Eu: *E. undulosus*, Ep: *E. poecilonotus*, Ec: *E. costae*; Em: *E. marginatus*; Ar: *A. regius*; Ln: *L. niloticus;* Pa: *P. americanus*. G: fish caught in Greek seas, I: imported in Greek markets, M: fish caught in the Mediterranean Sea.

To identify a gene region where *E. aeneu*s could form a distinct group, the *ND2* gene was analyzed. The melting region depicted in Figure 6a was manually adjusted to maximize the percent confidence for all samples, as illustrated in Figure S1d. This analysis also included *E. marginatus* samples, which exhibit high variability in this region due to the presence of various haplotypes among the imported specimens. The results demonstrated that all *E. aeneus* samples—whether from Greek catches, the Mediterranean Sea, or imported specimens from the Atlantic Ocean—were grouped into a single distinct cluster, separate from all other species (Figure 6b). In contrast to the *cytb* analysis, where variability was observed, the *ND2* gene successfully grouped all *E. aeneus* samples into a single, cohesive cluster.

From the four gene regions used in HRM analysis, only two (*cytb* and *ND2)* proved effective for accurately distinguishing *E. aeneus* from other species. However, these regions were unable to differentiate samples from Greek catches from other *E. aeneus* specimens originating from the Mediterranean Sea or the Atlantic Ocean.

#### 3.4.2. HRM Analysis for the Detection of *E. marginatus* Species

**Figure 7:**
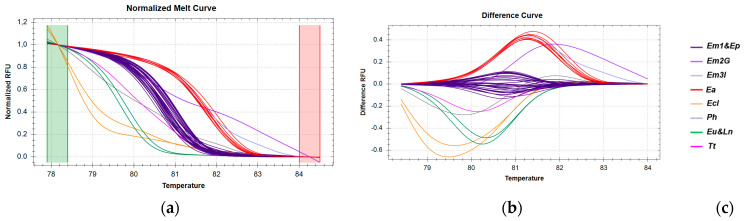
HRM reactions for the discrimination of *E. marginatus* from Greek fisheries compared to other species sold in the Greek market using the *16S rRNA* fragment: (**a**) Normalized melt curves from HRM analysis; (**b**) Difference curves from HRM analysis; (**c**) Species in clusters; Em: *E. marginatus*, Ea: *E. aeneus*, Eu: *E. undulosu*s, Ep: *E. poecilonotus*, Ec: *E. costae,* Ln: *L. niloticus,* Tt: *T. thynnus*, Ph: *P. hypophthalmus*; G: fish caught in Greek seas, I: imported in Greek markets.

The temperature range for 16S rRNA analysis used to discriminate *E. marginatus* was automatically adjusted (Figure 7a), resulting in percent confidence levels ranging from 95% to 100% (Figure S2a). Control samples that clustered within the *E. marginatus* (Em) group but with lower percent confidence levels (92–95%) were excluded from the analysis. Samples of *E. marginatus* from all geographic regions were grouped into three distinct clusters (Figure 7b), consistent with sequencing results. However, the *E. poecilonutus* sample was erroneously clustered with the Em1 group with a high percent confidence (97.2%).

**Figure 8:**
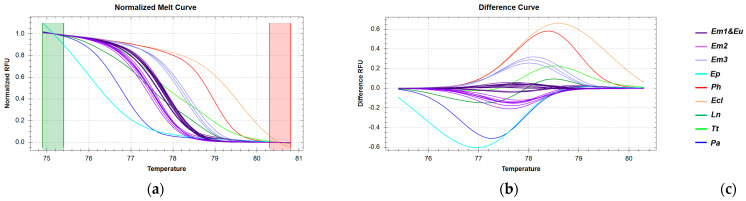
HRM reactions for the discrimination of *E. marginatus* from Greek fisheries compared to other species sold in the Greek market using the CR fragment: (**a**) Normalized melt curves of HRM analysis; (**b**) Difference curves HRM analysis; (**c**) Species in clusters; Em: *E. marginatus*, Eu: *E. undulosu*s, Ep: *E. poecilonotus*, Ec: *E. costae*, Ln: *L. niloticus*, Tt: *T. thynnus*, Ph: *P. hypophthalmus*, Pa: *P. americanus*; I: imported in Greek markets.

The temperature range for CR amplicon analysis used to discriminate *E. marginatus* was automatically adjusted (Figure 8a), resulting in percent confidence levels ranging from 95% to 100% (Figure S2b). Analysis of the melting curves reveals that all *E. marginatus* samples cluster into three distinct groups due to sequence variations within this region Figure 8b). These clusters are generally well-separated from other species, with the exception of *E. undulosous*, which clusters with the Em1 group. Although *P. hypophthalmus* forms a distinct cluster, the percent confidence level is low, likely due to insufficient levels of the PCR product.

**Figure 9:**
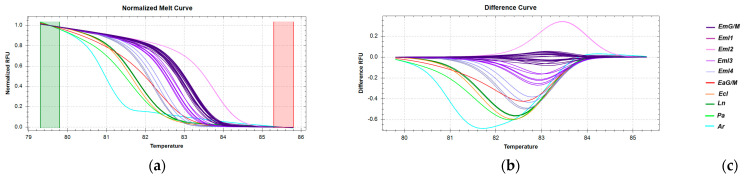
HRM reactions for the discrimination of *E. marginatus* from Greek fisheries compared to other species sold in the Greek market using the *ND2* fragment: (**a**) Normalized melt curves from HRM analysis; (**b**) Difference curves from HRM analysis; (**c**) Species in clusters; Em: *E. marginatus*, Ea: *E. aeneus*, Ec: *E. costae*, Ln: *L. niloticus*, Pa: *P. americanus*, Ar: *A. regius*; G: fish caught in Greek seas, M: fish caught in the Mediterranean Sea, I: imported in Greek markets.

For the *ND2* fragment analysis, all parameters were automatically configured using the software (Figure 9a), and clustering demonstrated very high confidence levels (95.4–99.9%) for *E. marginatus* samples (Figure S2c). Consistent with sequencing results, samples from Greek catches and the Mediterranean Sea were grouped into a single cluster, while imported fish from the Atlantic Ocean were separated into four distinct clusters, attributed to different SNPs. Furthermore, all *E. marginatus* clusters were clearly distinct from those of other analyzed species (Figure 9b,c).

Of the three gene regions used in HRM analysis for *E. marginatus*, only the *ND2* region effectively distinguished *E. marginatus* from all other species examined. Specifically, *ND2* analysis differentiated *E. marginatus* samples from Greek catches and the broader Mediterranean Sea from specimens originating the Atlantic Ocean. This suggests that the *ND2* region exhibits sufficient sequence variation to resolve geographical differences within *E. marginatus* populations, separating those found in Greek waters from others in the Mediterranean and Atlantic.

## 4. Discussion

The species selected for this study, *E. aeneus* and *E. marginatus*, are high-value fish widely traded and consumed in Greek fish markets and restaurants. Due to their large size, these species are often sold as fillets. Additionally, many imported fish, both fresh and frozen, are readily available in the Greek market. Morphological alterations caused by slicing, icing, or cooking make species identification impossible for consumers. As a result, incidents of food fraud have been reported involving these species [12,13,14,19]. This underscores the critical need for molecular methods of species identification. DNA sequencing-based techniques, such as DNA barcoding, serve as indispensable tools for achieving high accuracy and confidence in species verification as shown in many studies [50,54,55,56,57]. A large number of barcodes are available in the GenBank and Bold databases, but only a few are from the Greek seas. In this study, samples from the Greek seas were sequenced, and barcoding revealed a high degree of identity with samples from the Mediterranean and the Atlantic Sea. In the case of *E. aeneus*, two imported frozen samples labelled as *E. aeneus* were identified as *E. undulosus* and *E. poecilnotus*, two species that are not found in the Mediterranean Sea. Additionally, two samples served in a restaurant as *E. aeneus* were found to be *A. regius*, a fish commonly used in Greek aquaculture and sold at a significantly lower price. No incidents of mislabeling were found for *E. marginatus*.

DNA sequencing is accurate. However, it is costly and time consuming, and incidents of mislabeling in combination with the high demand of these fish by consumers raises the need for extensive market control, including the monitoring the geographic origin of the specimens. In this study, high-resolution melting analysis was used to discriminate between each one of the selected species when compared to different species or samples of the same species but of different geographic origins found in Greek market. This approach uses optimized species-specific reactions, enabling a clear positive or negative identification of an unknown sample belonging to the species under study, which are used as controls. Four mitochondrial gene regions were selected in the analyses that have different evolutionary rates: the highly conserved *cytb* and *16S* rRNA and the less conserved *ND2* and control region [58]. Fresh, frozen, and cooked samples of known identity were used as reference clusters to estimate the sensitivity, tolerance, efficiency, and consistency of each studied region and finally select the most suitable fragment. When studying frozen, processed commercially available, and cooked samples, DNA integrity is very important for the analysis; therefore, different isolation methods were used to produce high-quality DNA. The analysis proved equally effective for both frozen and cooked samples. Processes such as boiling, frying, adding sauces, or undergoing extended cycles of freezing and thawing did not affect the amplification of the tested mtDNA regions. Additionally, the clustering of each species, characterized by distinct peaks for each reference species, remained consistent regardless of the tissue type, its condition, or the DNA extraction method used. The clustering of each species showing characteristic peaks for each reference species was consistent regardless of the tissue used, its state, or the DNA extraction protocol. Also, shorter DNA regions (114–129 bp) were selected to ensure amplification even if DNA is degraded. Analysis parameters were either automatically selected or adjusted to ensure enough baseline data over at least a 10 °C degree window centered around the observed T_m_ of the amplified product [47]. The parameters were carefully selected to maximize the relative probability of a sample being in a cluster (percent confidence level) in the analyzed curves.

An effective marker for species authentication should exhibit variability even among closely related species while showing minimal intra-specific variation across its geographic range [59]. The analysis of the results showed that *16S rRNA* and CR were not suitable for the identification of *E. aeneus* and *E. marginatus*, as other species group within their clusters. The *16S* rRNA and CR region alignments show high variability between the studied species, also including single base gaps and very low percent identity with the reference sequences. As a result, false clustering could be attributed to the similar Tm values of different sequences due to length variation. Another possible explanation for the limitation of discriminating these samples is the fact that SNPs with reciprocally compensatory effects on nucleotide composition may neutralize each other, resulting in no significant impact on the shape of the melting curve [60]. As a result, in some cases, samples of two other species can be grouped in one cluster, thereby hiding the identity of the sample. Additionally, there is a possibility that the formation of secondary structures in the rRNA and CR regions could affect the accurate clustering of the samples.

In the analysis of *cytb*, *E. aeneus* samples were grouped in three different clusters for the species due to the presence of different SNPs, but these were distinct from other species clusters. The *ND2* fragment clearly distinguishes *E. aeneus* from the other species used in the analysis. However, one limitation encountered for *E. aeneus* samples in all analyses was that the differentiation of samples from different geographic origins, i.e., samples from Greek catches and samples from the Mediterranean Sea or the Atlantic Ocean, was not possible in all tested amplicons. When the analysis of the *ND2* region was performed for *E. marginatus*, all samples from the Mediterranean Sea clustered together, but samples from all other geographical loci presented different SNPs that lead to the grouping of these samples in different clusters. If the selected region has a fast evolutionary rate and a region accumulates mutations, one of the limitations of HRM analysis is the possibility that another unexpected SNP interferes with the accurate identification of the species [61]. In fact, alignment of the *ND2* region used in the analysis of samples from the Mediterranean with the *E. marginatus* mitochondrion complete genome (NC_087998.1) sampled in Brazil shows 100% identity, a finding that would classify this sample as Mediterranean. However, experimental proof of this hypothesis is not available. The limitation observed in the *ND2* analysis of *E. marginatus* samples was that the tested amplicon could distinguish samples from Greek catches from those originating elsewhere in the Mediterranean Sea.

High-resolution melting (HRM) analysis was effectively used to rapidly detect species substitutions of *Epinephelus* in the Greek market. All mislabeled samples that were accurately identified using sequencing were also successfully distinguished using HRM analysis, with the *ND2* amplicon providing the most reliable results. While HRM analysis does not directly identify the species involved in mislabeling, it significantly reduces the need for time-consuming and expensive sequencing by narrowing the focus to a small number of samples that do not cluster as expected. Therefore, direct sequencing is only necessary to verify HRM results for unknown samples that exhibit unexpected melting curve profiles.

Several methodologies have been used for the identification of *Epinephelus* species and the detection of fraudulent events [17,62,63,64,65]. In recent years, HRM analysis has been increasingly utilized for identifying fish species in Sparidae [24], Gadidae [66,67], hake [68], Takifugu pufferfish [69], Macrourus [27], sharks [70], pangasius [28], featherbacks [71], and salmonids [72]. Most of these studies focus on the identification of species but not always on the geographic origin of the samples. This methodology is accurately applicable to species identification. The ability for the method to be effective on frozen and cooked samples provides a significant advantage for extensive market control. However, despite their effectiveness in species-level discrimination, the *cytb* and *ND2* regions did not exhibit enough intra-species variability to differentiate *E. aeneus* or *E. marginatus* samples from different geographic origins. This limitation suggests that while these gene regions are suitable for inter-species differentiation, they may lack the resolution needed to detect population-level genetic differences within *E. aeneus*. Further analysis with additional markers or more variable regions may be required to resolve these finer-scale geographic distinctions.

## 5. Conclusions

The populations of several key Mediterranean fish species, particularly *E. aeneus* and *E. marginatus*, are declining, emphasizing the urgent need for sustainable fishing practices and effective conservation of fish stocks. To empower consumers to make informed choices and safeguard their health, it is vital to provide transparent, accurate, and comprehensive information about the species and origin of fish products. Ensuring the authenticity and traceability of fish products not only safeguards consumers but also supports the monitoring and conservation of vital fish stocks [73]. In this study, we employed high-resolution melting (HRM) analysis to identify the species of two *Epinephelus* species. This technique enables the analysis of a large number of samples within three hours in a single reaction using a single piece of equipment, making it ideal for implementation in multiple control laboratories across the supply chain. Our results demonstrate that HRM analysis is a highly effective and rapid method for accurately determining fish species, making it a valuable tool for preventing mislabeling and combating fraudulent practices in the seafood industry.

## Figures and Tables

**Table 1:** List of samples used for molecular identification. CM: Chios-Mytilene, CR: Crete, CY: Cyclades, DO: Dodecanese, IO: Ionian Sea, NA: North Aegean Sea, SG: Saronic Gulf, SP: Sporades, TG: Thermaikos Gulf, ME: Mediterranean Sea, IMP: imported in the Greek fish market sold as *E. aeneus* or *E. marginatus*; fish collected directly from local fish markets (FM), supermarkets (SM), or restaurants (RE);

Species	Sample Id	Site of Collection	FAO Region	Fresh Whole Fish	Frozen	Filleted	Cooked
*E. aeneus* (Ea)	EaG	CY	37.3.1	10	1 *		3 *
	EaG	SP	37.3.1	11	1 *		3 *
	EaG	SG	37.3.1	6	2 *		2 *
	EaG	DO	37.3.1	5	2 *		2 *
	EaG	CR	37.3.1	3	1 *		1 *
	EaG	TG	37.3.1	1			1 *
	EaG	CM	37.3.1	1			1 *
	EaG	IO	37.2.2	5	2 *		3 *
	EaM	ME	37.1.1	1			
	EaGM	FM/SM	37.1.1	1		1	
	EaI	IMP: FM/SM	37.1.3, 34.3.1, 34.3.2, 51, 57	3	3		4 *
	EaR	RE		1			6
*E. marginatus* (Em)	EmG	CY	37.3.1	5			2 *
	EmG	SP	37.3.1	2			2 *
	EmG	SG	37.3.1	17			5 *
	EmG	DO	37.3.1	2			1 *
	EmG	CR	37.3.1	8			3 *
	EmG	NA	37.3.1	1			1 *
	EmG	CM	37.3.1	1			
	EmG	IO	37.2.2	2			
	EmGM	FM/SM	37.1.1			2	
	EmM	ME	37.1.1	3			1
	EmI	IMP: FM/SM	37.1.3, 34.3.1, 34.3.2	8			4 *
	EmR	RE		2			2
*Epinephelus costae* (Ec)	EmG	SP	37.3.1	3			
	EmG	SG	37.3.1	4			
	EmG	CR	37.3.1	3			
	EmI	IMP: FM/SM	IMP: 37.1.3, 34.3.1, 34.3.2	6			3 *
	EmGM	FM/SM	37.1.1	1			
	EmR	RE		1			1
*L. niloticus* (Ln)	Ln	IMP: FM/SM			3	1	2 *
*P. americanus* (Pa)	Pa	IMP: FM/SM		1		1	1 *
*P. hypophthalmus* (Ph)	Ph	IMP: FM/SM				2	1 *
*T. thynnus*	Tt	IMP: FM/SM		1			

* samples/out of the total number in row that were cooked or frozen for the purposes of this study.

**Table 2:** PCR conditions for mitochondrial regions of *Epinephelus* and other species used in this study.

mtDNA Region	Primer Name	Primer Sequence	Annealing T	Fragment Length (bp)
*COI* barcode	m*COI*Fu ^1^	TCAACYAATCAYAAAGATATYGGCAC	55 °C	706
	m*COI*Ru ^1^	AGACTTCTGGGTGNCCAAARAATCA		
*16S rRNA*	*16s*F2 ^2^	AGTATGRGCGACAGAAAAGGA	53 °C	1389
	*16s*R1_L ^3^	TGCACCATTRGGATGTCCTGATCCAACATC		
*cytb*	GluF ^3^	AACCACCGTTGTTACTCAAC	53 °C	1141
	*cytb*UR ^3^	GCAAATAGGAARTATCAYTCRGG		
*12S rRNA*	12sF2 ^4^	AGCTAGACTTACACATGCAAGT	53 °C	668
	12sRe ^4^	CCATACGCTACACCTCGACC		
*ND2*	*ND2*Fe ^4^	CACTGACTACTTGCCTGAATAGG	56 °C	746
	*ND2*Re ^4^	GTCTTGTTTTGTTAGTTCTTGGAG		
*ND5*	ND5F ^4^	ACACCGGTCTCTGCCCTACT	52 °C	487
	ND5R ^4^	AGGGCTCAGGCGTTTAGGT		
CR	CRFea ^4^	CCCCTCAAGTACTCAAAGAG	52 °C	1103 *
	Perc12s1R ^5^	GCGGATACTCGCATGTGTAA		
CR	CRFea ^4^	CCCCTCAAGTACTCAAAGAG	52 °C	1103–1493 **
	CRR3e ^4^	GGATTGTTAAAGACTTCAAGGG		

^1^ modified FISHCO1LBC and FISHCO1HBC [35]; ^2^ primer from [26]; ^3^ modified from [36]; ^4^ this study; ^5^ primer from [37]; * only for *E. aeneus*; ** only for *E. marginatus.*

**Table 3:** Primers used for HRM analysis of mtDNA fragments.

GENE	Name	Primer	Annealing T/°C	Fragment Length (bp)
*16S rRNA*	q16sFe	AAGACGAGAAGACCCTATGGAG	56	114 bp
	q16sRe	TCGCCCCAACCAAAGACATTAG		
*cytb*	HcytEaF	TATCTGTATCTACGCCCACA	52	129 bp
	HcytEaR	GGAAGAACATATCCCACGAA		
*ND2*	HND2EmF	CTATTCTTGCCCTCTCCCTA	54	127 bp
	HND2EmR	GCGAAGGGTGCTAATTTTTG		
CR	qCRFe	ATGCACAGTAAGAACCTACCAA	54	124 bp
	qCRR2e	CTGAAATAGGAACCAGATGCCA		

**Table 4:** Accession numbers for PCR products sequenced in this study and the percent identity with *E. aeneus* and *E. marginatus* sequences from Genbank with 100% query cover.

mtDNA Region	Species	Accession Number	Percent Identity
*COI* barcode	*E. aeneus*	OR056374	100%
*COI* barcode	*E. aeneus*	OR056375	100%
*COI* barcode	*E. aeneus*	OR056376	99.85%
*COI* barcode	*E. aeneus*	OR056377	99.85%
*COI* barcode	*E. aeneus*	OR056378	100%
*COI* barcode	*E. aeneus*	OR056379	100%
*COI* barcode	*E. aeneus*	OR056380	100%
*COI* barcode	*E. aeneus*	OR056381	99.85%
*COI* barcode	*E. aeneus*	OR056382	100%
*COI* barcode	*E. aeneus*	OR056383	99.85%
*COI* barcode	*E. marginatus*	OR056425	99.85%
*COI* barcode	*E. marginatus*	OR056426	99.85%
*COI* barcode	*E. marginatus*	OR056427	99.85%
*COI* barcode	*E. marginatus*	OR056428	99.85%
*COI* barcode	*E. marginatus*	OR056429	99.70%
*COI* barcode	*E. marginatus*	OR056430	99.85%
*COI* barcode	*E. marginatus*	OR056431	99.70%
*COI* barcode	*E. marginatus*	OR056432	99.85%
*COI* barcode	*E. marginatus*	OR056433	99.70%
*COI* barcode	*E. marginatus*	OR056434	99.70%
*16S rRNA*	*E. aeneus*	OR091278	100%
*16S rRNA*	*E. marginatus*	OR091274	100%
*16S rRNA*	*E. marginatus*	OR091275	99.69%
*cytb*	*E. aeneus*	OR100408	99.73–100%
*cytb*	*E. aeneus*	OR100409	99.60–99.87%
*cytb*	*E. aeneus*	OR100410	99.60–99.87%
*cytb*	*E. aeneus*	OR100411	99.56–100%
*cytb*	*E. marginatus*	OR100404	99.42–100%
*cytb*	*E. marginatus*	OR100405	99.42–100%
*cytb*	*E. marginatus*	OR100406	99.26–99.85%
*cytb*	*E. marginatus*	OR100407	99.38–100%
*ND2*	*E. aeneus*	PQ824233	99.85%
*ND2*	*E. aeneus*	PQ824234	100%
*ND2*	*E. aeneus*	PQ824235	99.85%
*ND2*	*E. aeneus*	PQ824236	99.70–99.85%
*ND2*	*E. aeneus*	PQ824237	99.85–100%
*ND2*	*E. aeneus*	PQ824238	99.85–100%
*ND2*	*E. aeneus*	PQ824239	99.70–99.85%
*ND2*	*E. marginatus*	PQ824240	99.85%
*ND2*	*E. marginatus*	PQ824241	99.85%
*ND2*	*E. marginatus*	PQ824242	99.85%
*ND2*	*E. marginatus*	PQ824243	99.85%
*ND2*	*E. marginatus*	PQ824244	99.71%
*ND2*	*E. marginatus*	PQ824245	99.71%
*ND2*	*E. marginatus*	PQ824246	99.71%
*ND2*	*E. marginatus*	PQ824247	99.85%
*ND2*	*E. marginatus*	PQ824248	99.85%
*ND2*	*E. marginatus*	PQ824249	99.85%
CR	*E. aeneus*	PQ852100	99.84%
CR	*E. aeneus*	PQ852101	99.53%
CR	*E. aeneus*	PQ852102	99.69%
CR	*E. aeneus*	PQ852103	99.53%
CR	*E. aeneus*	PQ852104	99.84%
CR	*E. aeneus*	PQ852105	99.52%
CR	*E. aeneus*	PQ852106	99.53%
CR	*E. aeneus*	PQ852107	98.91%
CR	*E. aeneus*	PQ852108	99.15%
CR	*E. aeneus*	PQ852109	99.53%
CR	*E. marginatus*	PQ852110	98.18%
		PQ852111	98.18%

**Table 5:** Optimized analysis parameters for HRM analysis of mitochondrial gene regions.

Gene	Species	Temperature Shift (°C)	Melt Curve Shape Sensitivity (%)	Tm Difference Threshold (°C)
*16S rRNA* *	Ea	0.30 *	30 *	0.50
*16S rRNA* *	Em	0.30 *	50	0.20 *
*cytb*	Ea	0.20	50	0.20 *
CR	Ea	0.20	50	0.30 *
CR	Em	0.20	50	0.30 *
*ND2*	Ea	0.20	50	0.15
*ND2*	Em	0.20	50	0.20 *

* Adjusted manually.

## Data Availability

The data presented in this study are available in the GenBank NIH genetic sequence database under the following accession numbers: OR056374-OR056383, OR056425-OR056434, OR091278, OR091274- OR091275, OR100408- OR100411, OR100404- OR100407, PQ824233-PQ824239, PQ824240-PQ824249, PQ852100-PQ852109, PQ852110, and PQ852111.

## Supplementary Materials

The following supporting information can be downloaded at: https://www.mdpi.com/article/10.3390/genes16030255/s1, Figure S1: Maximum confidence percent values for *E. aeneus* obtained from HRM analysis; Figure S2: Maximum confidence percent values for *E. marginatus* obtained from HRM analysis.
