# Supplementary material for: Rapid Detection of Epinephelus Species Substitution in the Greek Market Using High-Resolution Melting Analysis
Source: Genes (Basel). 2025 Feb 22;16(3):255. doi: 10.3390/genes16030255 (PMC11942476; doi:10.3390/genes16030255)
Supplement: Supplementary file 1 [file genes-16-00255-s001.zip › SM1.pdf]

Epinephelus aeneus 16s	
Cluster	Percent Confidence
Ea	98,3
Ea	99,1
Ea	98,7
Ep	97,8
Pa	68,5
Ea	98,7
Ea	98,7
Ea cooked	98,9
Ea	99,7
Ea	99,9
Ea	99,5
Ea	99,9
Ea	98,9
Ea	98,2
Ea	95,6
Ea	99,7
Ea	99,9
Ea	99,5
Ea	99,6
Ea	99,1
Ea cooked	99,3
Ea	99,3
Ea	99,8
Ea	99,9
Ea	99,3
Ea	98,7
Ea	99,5
Ea	99,7
Ea	99,6
Ea	98,8
Ea	99,6
Ea	98,8
Ea cooked	98,7
Ea	99,1
Ea	98,9
Ea	98,5
Ea	99,5
Ea	99,0
Eu and Ln	99,4
Eu and Ln	99,5
Tt	100,0

Epinephelus aeneus cytb	
Cluster	Percent Confidence
Ar and Ep cooked	98,6
Ar and Ep	96,8
Ar and Ep	97,0
Ea1	98,5
Ea1	99,5
Ea1	99,0
Ea1 cooked	99,0
Ea1	99,9
Ea1	95,2
Ea1	99,3
Ea1	99,4
Ea1	98,2
Ea1	97,8
Ea1	99,7
Ea1	98,4
Ea1	98,3
Ea2	98,1
Ea2	97,5
Ea2	92,0
Ea2	98,7
Ea2	99,1
Ea2 cooked	92,8
Ea2	98,3
Ea2	97,3
Ea2	97,9
Ea3	98,0
Ea3	94,4
Ea3	99,2
Ea3	99,9
Ea3	98,5
Ea3 cooked	99,9
Ea3	98,5
Ec	99,3
Eu	98,8
Ln	100,0
Pa	91,6
Ph	98,7

Epinephelus aeneus CR	
Cluster	Percent Confidence
Ea1 cooked	98,5
Ea1	97,3
Ea1	96,9
Ea1	98,3
Ea1	98,0
Ea1	98,0
Ea1	98,5
Ea1	98,2
Ea1 Cooked	99,0
Ea1	97,8
Ea1	98,5
Ea1	98,8
Ea1	99,2
Ea1	99,2
Ea1	99,6
Ea1	98,9
Ea1	97,5
Ea1	99,7
Eu	99,3
Ea1	99,6
Ea1	100,0
Ea1	98,3
Ea1	99,6
Ea1	99,4
Ea1	98,3
Ea1	99,7
Ea1	98,7
Ea1	98,1
Ea2	97,0
Ea2	99,1
Ea2 cooked	98,1
Ea2	99,7
Ec	99,7
Ep	100,0
Ln	100,0
Pa	98,0
Ph	80,2

Epinephelus aeneus ND2	
Cluster	Percent Confidence
Ar	100,0
EaG/M	99,8
EaG/M	99,3
EaG/M	99,2
EaG/M cooked	99,6
EcG	99,9
EcI	98,3
EmG/M	98,9
EmG/M	98,5
EmG/M cooked	99,8
EmG/M	99,7
EmG/M	99,5
EmG/M	99,8
EmG/M cooked	99,4
EmG/M	99,6
EmG/M	99,9
EmG/M	99,5
EmG/M	99,7
EmG/M	98,4
EmG/M	97,9
EmG/M	99,7
EmG/M	99,5
EmI1	98,6
EmI1 cooked	97,6
EmI1	99,6
EmI1	99,8
EmI2	98,8
EmI2	98,8
EmI3 cooked	97,8
EmI3	98,0
EmI4	95,7
EmI4	97,9
Ep	100,0
Eu	98,8
Ln	99,0
Ln	98,6
Pa	95,6

(a) (b) (c) (d)

Figure S1. Maximum Confidence Percent values indicating the relative probability of a sample being grouped in a cluster parametrized for the discrimination of *E. aeneus*. Fish species is indicated for fresh and frozen samples and Melt curve shape sensitivity and the Tm difference threshold values are given in 3.4 for each fragment: (a) *16s*; (b) *cytb*; (c) CR; (d) *ND2*. Fish species is indicated for fresh and frozen samples; Ea: *Epinephelus aeneus*, Em: *Epinephelus marginatus*, Eu: *Epinephelus undulosus*, Ep: *Epinephelus poecilonotus*, Ec: *Epinephelus costae*, Ar: *Argyrosomus regius*; Ln: *Lates niloticus*, Ph: *Pangasianodon hypophthalmus*, Tt: *Thunnus thynnus*, Pa: *Polyprion americanus*; the indication "cooked" is added for cooked or processed samples.
