# Supplementary material for: Rapid Detection of Epinephelus Species Substitution in the Greek Market Using High-Resolution Melting Analysis
Source: Genes (Basel). 2025 Feb 22;16(3):255. doi: 10.3390/genes16030255 (PMC11942476; doi:10.3390/genes16030255)
Supplement: Supplementary file 1 [file genes-16-00255-s001.zip › SM2.pdf]

Epinephelus marginatus 16s	
Cluster	Percent Confidence
Ea cooked	98,1
Ea	96,0
Ea	98,9
Ea	97,3
Ea	98,1
Ea	95,0
Ea	96,8
Ea	95,9
Ecl	99,9
Ecl2	100,0
Em1 cooked	96,4
Em1	99,6
Em1	99,0
Em1	96,2
Em1	98,6
Em1	97,6
Em1	96,6
Em1	97,2
Em1 cooked	97,3
Em1	98,3
Em1	96,0
Em1	98,5
Em1	98,2
Em1	96,8
Em1 cooked	96,9
Em1	96,6
Em1	98,7
Em1	96,5
Ep	97,2
Em1	96,4
Em1	98,6
Em1	97,9
Em1	97,2
Em1	96,9
Em1	98,0
Em1	98,7
Em1	97,9
Em1	96,1
Em1	95,9
Em1	95,7
Em1	97,8
Em1	99,1
Em2	98,1
Eu	99,1
Ln	99,2
Ph	98,1
Tt	95,6

(a)

Epinephelus marginatus CR	
Cluster	Percent Confidence
Em1	99,1
Em1	99,1
Em1 cooked	97,6
Em1	98,9
Em1	95,5
Em1	97,2
Em1	98,9
Eu	98,5
Em1	96,1
Em1	97,6
Em1	98,7
Em1	96,8
Em1	97,9
Tt	98,9
Ph	51,6
Pa	93,9
Ln	98,9
Ep	100,0
Em3	99,0
Em3 cooked	96,9
Em3	95,7
Em2	98,0
Em2	98,8
Em2	99,5
Em2	96,6
Em2	99,0
Em2 cooked	98,7
Em2	96,9
Ecl	100,0

(b)

Epinephelus marginatus ND2	
Cluster	Percent Confidence
Ar	100,0
Ea	99,7
Ecl	95,4
EmG/M	99,5
EmG/M	98,1
EmG/M	99,5
EmG/M	99,8
EmG/M	99,9
EmG/M	99,0
EmG/M	99,8
EmG/M	99,9
EmG/M	99,8
EmG/M	99,8
EmG/M	99,5
EmG/M	99,4
EmG/M	99,6
EmG/M	99,9
EmG/M cooked	99,5
EmG/M	99,3
EmG/M	98,3
EmG/M	99,4
EmG/M	99,9
EmG/M cooked	99,7
Eml1	97,8
Eml1 cooked	98,3
Eml1	98,2
Eml1	99,7
Eml1	99,9
Eml1	96,8
Eml2	99,4
Eml2	99,6
Eml3 cooked	97,9
Eml3	98,2
Eml4 cooked	95,5
Eml4	98,2
Ln cooked	99,3
Ln	97,6
Pa	99,7

(c)

Figure S2. Maximum Confidence Percent values indicating the relative probability of a sample being grouped in a cluster parametrized for the discrimination of *E. marginatus*. Melt curve shape sensitivity and the Tm difference threshold values are given in 3.4 for each fragment: (a) *16s*; (b) *cytb*; (c) *ND2*. Fish species is indicated for fresh and frozen samples; Em: *Epinephelus marginatus*, Ea: *Epinephelus aeneus*, Eu: *Epinephelus undulosus*, Ep: *Epinephelus poecilonotus*, Ec: *Epinephelus costae*, Ar: *Argyrosomus regius*; Ln: *Lates niloticus*, Ph: *Pangasianodon hypophthalmus*, Tt: *Thunnus thynnus*, Pa: *Polyprion americanus*; the indication "cooked" is added for cooked or processed samples.
